# Neonatal Applications of Heliox: A Practical Review

**DOI:** 10.3389/fped.2022.855050

**Published:** 2022-03-10

**Authors:** Tomasz Szczapa, Patryk Kwapień, T. Allen Merritt

**Affiliations:** ^1^Department of Newborns' Infectious Diseases, Chair of Neonatology, Poznan University of Medical Sciences, Poznan, Poland; ^2^Department of Neonatology, Chair of Neonatology, Poznan University of Medical Sciences, Poznan, Poland; ^3^Division of Neonatology, Loma Linda University School of Medicine, Loma Linda, CA, United States

**Keywords:** heliox, helium, newborn, neonate, respiratory distress, respiratory failure, mechanical ventilation, non-invasive respiratory support

## Abstract

Heliox is a mixture of helium and oxygen that may be utilized as an alternative to air-oxygen during the ventilatory support in the neonate. Special physical properties of Heliox, particularly low density, allow for improved gas flow and diffusion. First reports of Heliox use in the pediatric population were published in 1930s; however, this therapy has never gained widespread popularity despite its described beneficial effects. Historically, this was largely due to technical challenges associated with Heliox ventilation that significantly limited its use and realization of large-scale clinical trials. However, nowadays several commercially available ventilators allow easy and safe ventilation with both conventional and non-invasive modes. In the era of minimally invasive respiratory interventions in the newborn Heliox could be seen as a therapy that may potentially decrease the risk of non-invasive ventilation failure. This review presents pathophysiologic rationale for the use of Heliox in the newborn, and summarizes available data regarding applications of Heliox in the setting of neonatal intensive care unit based on clinical studies and findings from animal models. Mechanisms of action and practical aspects of Heliox delivery are thoroughly discussed. Finally, future research directions for neonatal use of Heliox are proposed.

## Introduction

Management of respiratory failure remains one of the major challenges in neonatology. Increasing knowledge of the pathophysiology and technological advances allow continuous optimization of the ventilatory support. With a standard approach air and oxygen are blended during ventilation, however it is possible to use an alternative gas mixture of helium and oxygen–Heliox. It has been known for quite a long time that despite unique properties it did not gain widespread popularity (1, 2). The first attempts to use Heliox were reported in 1934 when Dr. Alvan Barach described his research in patients with asthma and airway obstruction (1, 2). Unfortunately, due to limited availability of the gas, dedicated equipment necessary for safe and effective delivery and relatively small number of studies, Heliox has not been used routinely in the Neonatal Intensive Care Units (NICU). Non-invasive respiratory support is usually preferred in neonates with respiratory support; however, a significant proportion of patients may fail the therapy (3, 4). In the era of minimally invasive interventions in the newborn, heliox potentially offers a therapy that may either by decreasing the risk of non-invasive ventilation failure or the risk of lung injury in infants requiring mechanical ventilation. In this review we summarize clinically relevant information regarding the properties of Heliox and its applications in the neonate.

## Physical Properties

**Figure 1:**
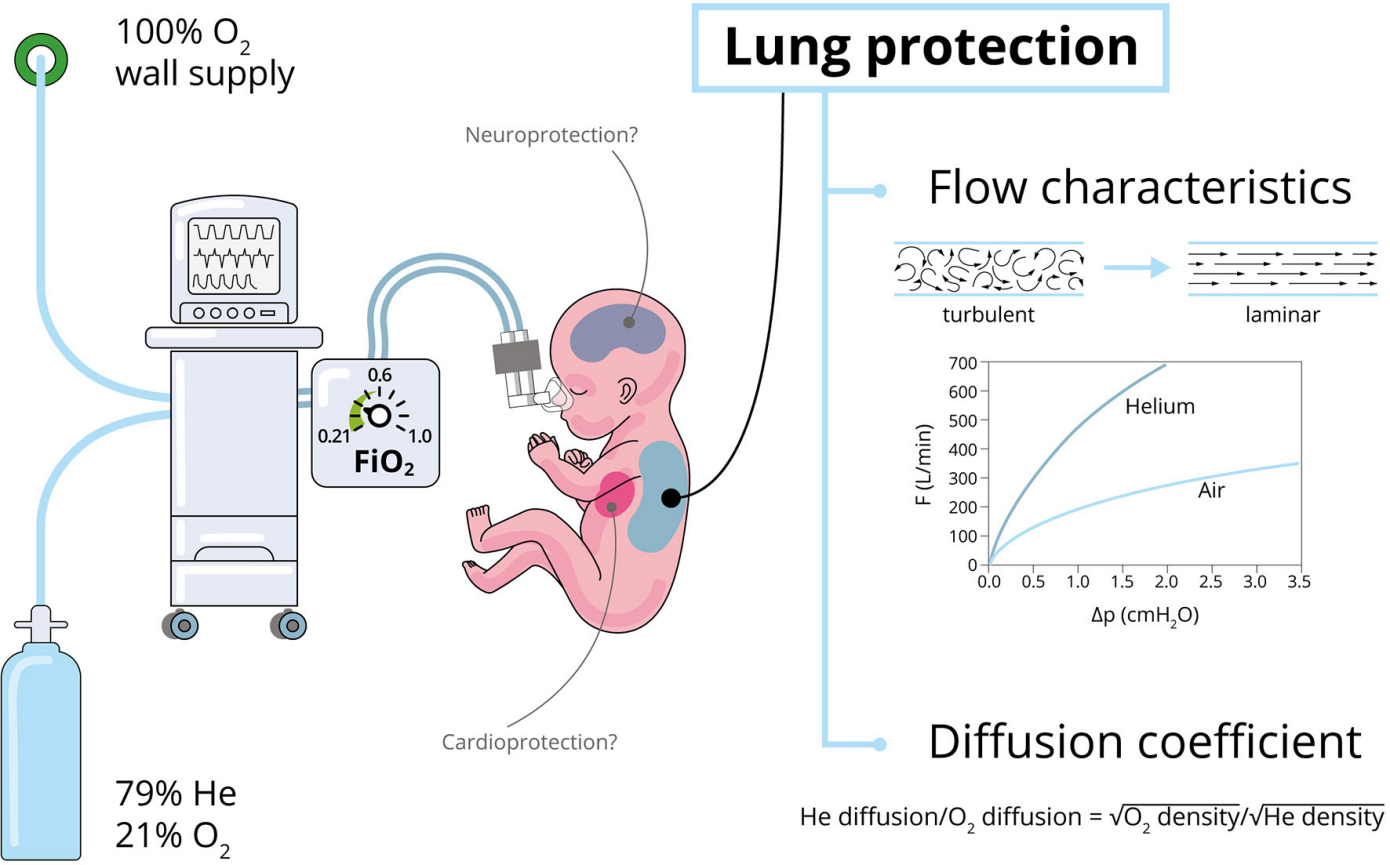
Properties and effects of Heliox (4, 6). Optimal FiO_2_ values to achieve the expected Heliox effects were marked with green on the blender (the lower the FiO_2_ the greater the effect).

Heliox is an odorless, non-explosive, non-flammable gas. Its density is about 3 times lower than the density of air influencing the therapeutic value of Heliox (Table 1). Due to the small cross-sectional diameter of the airways in the newborn the relatively high resistance (as compared to older patients) is an important factor influencing gas exchange. When Heliox is applied instead of air-oxygen mixture it reduces the turbulent flow through the airways and allows delivery of a given volume using a lower pressure (Figure 1) (4). This feature may help to both avoid lung barotrauma and improve ventilation in patients with increased airway resistance (4, 7). Effects of Heliox on flow characteristics depend on the fraction of inspired oxygen (FiO_2_)—it becomes less pronounced with decreasing He:O_2_ ratio and increasing density (4, 7).

**Table 1:** Physical properties of Heliox (5).

	**Heliox 21%/79%**	**Air**
Density (kg/m^3^) 37°C	0.389	1.139
Viscosity (η)(μP) 37°C	205.67	189.56
Diffusion index (cm^2^/s)	0.56	0.138
Thermal conductivity (μcal/cm/s/°K)	352	58

Another important effect of Heliox is explained by Graham's law—the rate of gas diffusion is inversely proportional to the square root of its mass density (Figure 1) (6). Hence, the use of helium-oxygen instead of air with an unchanged oxygen concentration in the inspired mixture can result in a better diffusion of oxygen and carbon dioxide in the alveoli (4). High thermal conductivity of helium may result in faster changes of tissue temperature which should be considered during the therapy (8, 9).

## Physiological Effects of Heliox

There are no confirmed toxic effects of helium-oxygen mixtures on the human body. It has been described as “biologically inert” by some authors; however, experimental studies have shown that helium exerts cellular effects *in vitro* and *in vivo* (10, 11). Long-term studies on newborn rabbits, maintained in specially designed Heliox-filled incubators (12), confirmed the safety of Heliox, finding no effect of the intervention on growth or other physiological parameters of the animals (13).

Helium-oxygen seems well-tolerated when administered with CPAP or positive pressure ventilation, provided that sufficient safety measures are taken (summarized in Section “Practical Issues”). The majority of studies report no significant effects on heart rate, peripheral capillary oxygen saturation or cerebral tissue oxygenation (14–19). Publications from the 1980s and 1990s described hypoxia during heliox administration to a plexiglass chamber or oxygen hood (20, 21). According to Butt et al. the decrease in transcutaneous partial pressure of oxygen (tcPO_2_) was observed when helium-oxygen was delivered *via* oxygen hood but not with CPAP. The authors speculated that the decrease of oxygenation was caused by a reduction in lung volume in relation to the decrease in expiratory resistance that “might even be considered as a sign of recovery” (20, 22). In contrast to other studies de Gamara et al. observed a small decrease in skin (but not rectal) temperature that could be associated with this mode of delivery (7).

Heliox ventilation was reported to reduce respiratory effort, diaphragmatic load and display protective effects against atelectasis and airway collapse. In addition, it facilitates the distribution of respiratory gases in narrow and/or constricted airways (17, 23–25). Helium-oxygen may allow better penetration into the peripheral parts of the lungs and improve ventilation/perfusion ratio. Heliox can also increase mixing of gases in the alveoli and improve CO_2_ elimination (8, 26). A decrease in the respiratory rate was observed when helium-oxygen was used instead of air-oxygen (16, 20). It was found that heliox ventilation is associated with a higher expiratory flow when compared to standard mixture at the same pressure (4, 14, 18). This may facilitate passive exhalation, offsetting the danger of gas trapping and the accompanying barotrauma (4, 7).

Several studies reported a decrease in the oxygen demand with Heliox ventilation. This may be beneficial in the preterm infants as they are particularly vulnerable to the toxic effects of oxygen. Additionally, increasing the He:O_2_ ratio may result in a greater improvement of flow characteristics (14, 15, 23, 27).

Helium-oxygen ventilation has been reported to result in significant decrease of serum inflammatory markers such as IL-6, IL-8, CRP, and TNFα in neonates with meconium aspiration (9). Similar findings were made in animal models. Heliox use was associated not only with a significant decrease of IL-8 and myeloperoxidase but also reduced muscle injury score of the diaphragm and better aeration of heliox-ventilated lungs (25, 27).

In a study carried out in preterm infants the electrical activity of the diaphragm (EDI) was compared during air-oxygen and heliox non-invasive respiratory support. It has been shown that EDI decreased significantly after heliox ventilation was started (17).

Cardioprotective and neuroprotective effects of helium-preconditioning were also reported. In a rat model inhalation of helium mixtures at concentrations ≥30% before cardiac ischaemia resulted in a reduction of the size of the infarct (28). It has been speculated that preconditioning with helium impacts cardiac mitochondrial function (11). Similarly, in rats with hypoxic ischaemia helium-preconditioning was associated not only with decreased infarction area but also with increased expression of antioxidant enzymes, less apoptosis and improved neurological outcomes. Proposed mechanism of action included induction of the production of nitric oxide and reduced inflammatory response (29).

## Clinical Applications of Heliox

### Respiratory Distress Syndrome

In the early 1990s a French group conducted a randomized controlled trial (RCT) on Heliox mechanical ventilation (MV) in neonates with respiratory distress syndrome (RDS). They found that Heliox vs. air-oxygen ventilation resulted in lower oxygen demand, shorter duration of MV, lower rates of bronchopulmonary dysplasia (BPD) and better survival (27). It is important to mention that this study was carried out when surfactant administration was not yet a standard of care and alternative methods of optimization of therapy were pursued. Two decades later the role of Heliox was investigated in infants with RDS requiring MV and increased oxygen concentration (FiO_2_ ≥ 0.4) despite surfactant administration. Heliox MV was associated with a significant increase in tidal volume and lower oxygen demand (14). Improvement in ventilatory support requirements (e.g., decrease in mean airway pressure) and gas exchange was described by Migliori et al. in in long-term mechanically ventilated very low birth weight infants (23).

The interest in Heliox recurres in an era of less invasive approach to respiratory support with the aim to decrease the risk of non-invasive ventilation failure has increased. Colnaghi et al. randomized preterm infants with RDS to receive nCPAP with helium-oxygen (4:1 He:O_2_) or medical air. Heliox significantly decreased the need for mechanical ventilation and surfactant (15). A Chinese RCT from 2014 reported the results of nasal intermittent positive pressure ventilation with Heliox in neonates with RDS. The therapy resulted in shorter ventilation and better CO_2_ removal (30). In a recent study by Neumann-Klimasinska et al. helium-oxygen mixture was applied with non-invasive neurally adjusted ventilatory assist (NIV-NAVA) as primary respiratory support or post-extubation. It was found that Heliox NIV-NAVA was associated with a prompt and significant reduction of Edi indicating reduced respiratory effort. Decreased respiratory rate and peak inspiratory pressure were also observed (17).

A systematic review and meta-analysis from 2016 assessed the effects of Heliox non-invasive ventilation (NIV) in preterm infants with RDS. It included 2 RCTs and 1 quasi-randomized controlled trial with a total number of 123 neonates. In comparison to standard gas mixture Heliox NIV significantly decreased the incidence of intubation (RR: 0.42; 95% CI: 0.23–0.78). Its use was also associated with reduction of PaCO_2_ (MD: −9.61; 95% CI: −15.76 to −03.45) and less frequent surfactant administration (RR: 0.25; 95% CI: 0.10–0.61). There were no significant differences among other secondary outcomes including the length of NIV and hospitalization, incidence of bronchopulmonary dysplasia (BPD), patent ductus arteriosus, necrotizing enterocolitis, intraventricular hemorrhage, periventricular leukomalacia and death (31).

### BPD

While ventilation with Heliox was associated with less frequent intubation, lower concentrations of inflammatory markers and lower oxygen demand it might be expected that the use of this mixture would result in reduced risk of BPD. Indeed, this was reported in the early RCT of Heliox MV by Ellau et al. but not confirmed by the metaanalysis regarding Heliox NIV (27, 31).

Helium-oxygen mixtures were also used in infants with established BPD: either non-invasively or *via* endotracheal tube. In a study by Wolfson et al. helium-oxygen mixtures (He:O_2_ ratios of 80:20 and 70:30) were administered in spontaneously breathing patients with BPD using face masks while pulmonary function parameters were monitored. In the clinical examination infants “appeared to breathe more regularly and with less effort” on Heliox vs. air-oxygen. This was in accordance with results of performed measurements—significant decrease of work of breathing and pulmonary resistance (25). On practical note: it seems important to stress the fact that beneficial effects of Heliox were observed in infants with BPD when Heliox was administered with positive pressure but not with plexiglass chamber or oxygen hood (20, 21).

Our group investigated the effects of Heliox in infants with severe BPD. MV with helium-oxygen mixture was well tolerated and associated with significant increase in dynamic compliance, peak expiratory flow rate (PEFR) and minute ventilation. Additionally, Heliox MV resulted in improved oxygenation and allowed significant reduction of FiO_2_. PaCO_2_ decreased during helium-oxygen administration but the difference was statistically insignificant (18).

### Meconium Aspiration Syndrome

Based on theoretical assumptions regarding the influence on helium on gas flow and diffusion and the pathophysiology of MAS that involves elevated pulmonary resistance and reduced lung compliance Heliox ventilation seems to offer benefits over ventilation with air-oxygen.

So far, only two clinical studies regarding Heliox use in MAS were published. First results were reported by our group in 2011. Heliox MV was found to significantly improve oxygenation index and alveolar-arterial oxygen tension difference. Observed increase in PEFR and decrease in PaCO_2_ were not significant.

A decade later Ma et al. presented results of a RCT that utilized the same ventilation mode (pressure-controlled synchronized intermittent mandatory ventilation) and device (Avea ventilator) but a longer time of Heliox delivery (1 vs. 6 h). Among primary outcomes PaO_2_/FiO_2_ was significantly higher in the neonates ventilated with helium-oxygen while their time to extubation and length of hospitalization were significantly shorter vs. the control group on air-oxygen. PaCO_2_ was significantly lower at 2–48 h after intervention. IL-6, IL-8, CRP, and TNF-α were significantly lower after 6 h of Heliox MV. Similar reduction was found for the markers of myocardial injury (creatine kinase and creatine kinase isoenzyme) after 24 h. There were no significant differences in the rate of pneumothorax or other complications between groups (10).

Severe MAS may be complicated by persistent pulmonary hypertension of the newborn (PPHN). In such scenario it seems that Heliox MV can be safely combined with inhaled nitric oxide (iNO). Combined use of these therapies described in a case report of a preterm infant with localized interstitial pulmonary emphysema and pulmonary hypertension resulted in full recovery. Chest X-ray after 5 h of Heliox MV revealed decreased air-trapping; significant improvement of oxygenation was also observed (14). Concurrent use of Heliox and iNO was also reported in infants with congenital diaphragmatic hernia (32).

### Bronchiolitis

Bronchiolitis is the main reason for hospitalization of infants in the developed countries and may lead to respiratory failure requiring intensive care (33). There are limited therapies recommended for routine use in patients with this clinical problem. Airway obstruction caused by mucus and oedema results in turbulent flow and increased resistance. Decreased lung compliance, increased end-expiratory pressure, air-trapping and ventilation/perfusion mismatch are additional problems observed in bronchiolitis. Based on these pathophysiological features and theoretical assumptions Heliox seems to address the needs of an infant with bronchiolitis very well (33, 34).

A Cochrane Database review from 2015 included 7 trials carried out in 447 infants <2 years with respiratory distress due to viral bronchiolitis. Only one study was performed in intubated patients (13 infants). All trials used different protocols for Heliox therapy; no adverse events were reported. Regardless of the utilized protocol helium-oxygen mixture administration resulted in significant reduction of mean clinical respiratory score in the first hour after starting treatment when compared to air/oxygen: MD −1.04 (95% CI −1.60 to −0.48, four trials, 138 infants, moderate quality evidence). Authors indicated that the outcome had statistical heterogeneity that could be explained by the wide differences in the baseline severity of disease between studies and limited number of patients in each trial. Heliox did not reduce the risk of intubation, rate of emergency department discharge or length of treatment for respiratory distress. However, in infants on nCPAP right from the start helium-oxygen reduced the length of treatment: MD −0.76 days (95% CI −1.45 to −0.08, one trial, 21 infants, low quality evidence) (35). Chowdury et al. concluded that “Heliox therapy does not reduce length of treatment unless given *via* a tight-fitting facemask or CPAP” (36). A study from 2019 confirmed the lack of effect of Heliox administered *via* low flow nasal cannula in patients with acute viral bronchiolitis (37). Similarly, to the observations from studies investigating the role of helium-oxygen mixtures in infants with BPD this finding highlights the role of Heliox administration with positive pressure (20, 21).

Another modality of respiratory support that was shown to be safe and more effective than standard oxygen therapy in infants with bronchiolitis is high flow nasal cannula (HFNC) (37, 38). Considering this fact and encouraging results of a study in which HFNC with Heliox utilized in animal models of lung injury resulted in reduced work or breathing and less lung inflammation it seems that helium-oxygen high flow therapy may be an interesting alternative to nCPAP (24). There was an attempt to assess Heliox HFNC in bronchiolitis in an American trial that was terminated in 2019 (NCT02373683). Hopefully, this promising therapy will be assessed in the future.

### Other Indications

Heliox ventilation can be considered in case of difficult airway or airway obstruction (e.g., foreign body aspiration, post-extubation laryngeal stenosis, and extrinsic obstruction of airways) (39). Available reports were summarized in Table 2.

**Table 2:** Clinical reports of Heliox utilization in neonatology.

**Indication**	**References**
Respiratory distress syndrome	(14, 15, 23, 27, 40)
Bronchopulmonary dysplasia	(18, 20, 21, 25)
Meconium aspiration syndrome	(10, 19)
Broncholitis	(7, 36, 41–43)
Pulmonary interstitial emphysema	(44, 45)
Congenital diaphragmatic hernia	(32)

Helium-oxygen may also optimize the delivery of nebulized drugs; however, its efficacy may depend on the mode of ventilation (46–48). In pediatric patients with asthma exacerbations Heliox-driven albuterol nebulization was found to result in better clinical effects (e.g., improved pulmonary index) as compared to 100% oxygen (49, 50). However, Bigham et al. reported no significant impact of this therapy on length of hospitalization or time to eligibility for intensive care unit discharge (51). Impact of Heliox on effectiveness of aerosol delivery was also assessed in pediatric *in vitro* models. In a model of MV (simulated lungs of 10 and 30 kg child, pressure-regulated volume-controlled ventilation) helium-oxygen increased albuterol delivery administered by metered-dose inhaler to the end of the endotracheal tube (48). Ari et al. studied aerosol delivery utilizing pediatric high flow nasal cannula and vibrating mesh nebulizer with Heliox vs. 100% oxygen. Drug deposition was similar at 3 L/min flow but significantly greater with Heliox at 6 L/min (46).

## Practical Issues

**Figure 2:**
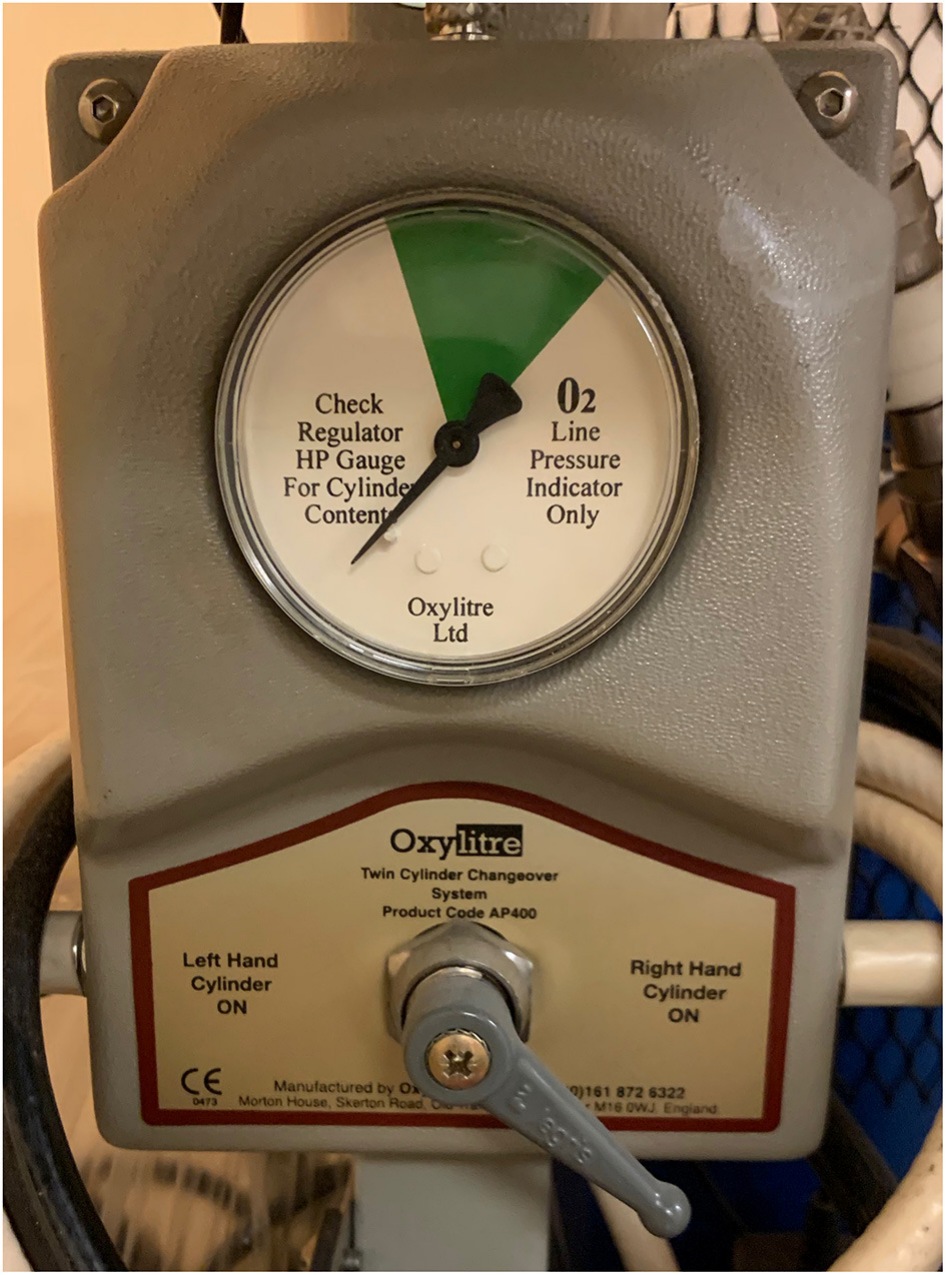
Cylinder changeover system.

Heliox administration is relatively easy provided that the personnel are aware of gas properties and uses appropriate setup with cautious monitoring. The pressurized gas is usually provided in cylinders of different sizes, hence for continuous supply it is necessary to secure an adequate stock of gas (ideally with a changeover system that allows smooth exchange of tanks, Figure 2). Estimated consumption may vary significantly and will depend on the mode of ventilation, settings and device. Based on Authors' personal experience—one cylinder of Heliox using the same mode and settings with Servo-I (Getinge, Sweden) allows longer ventilation than Avea (Vyaire, USA) ventilator. Similarly, Berkenbosch et al. observed that an older model of Servo ventilator (Servo 300, Siemens, Germany) had lower rates of consumption then other devices. It was also reported that gas consumption was greater during pressure-controlled vs. volume-controlled ventilation (52).

**Figure 3:**
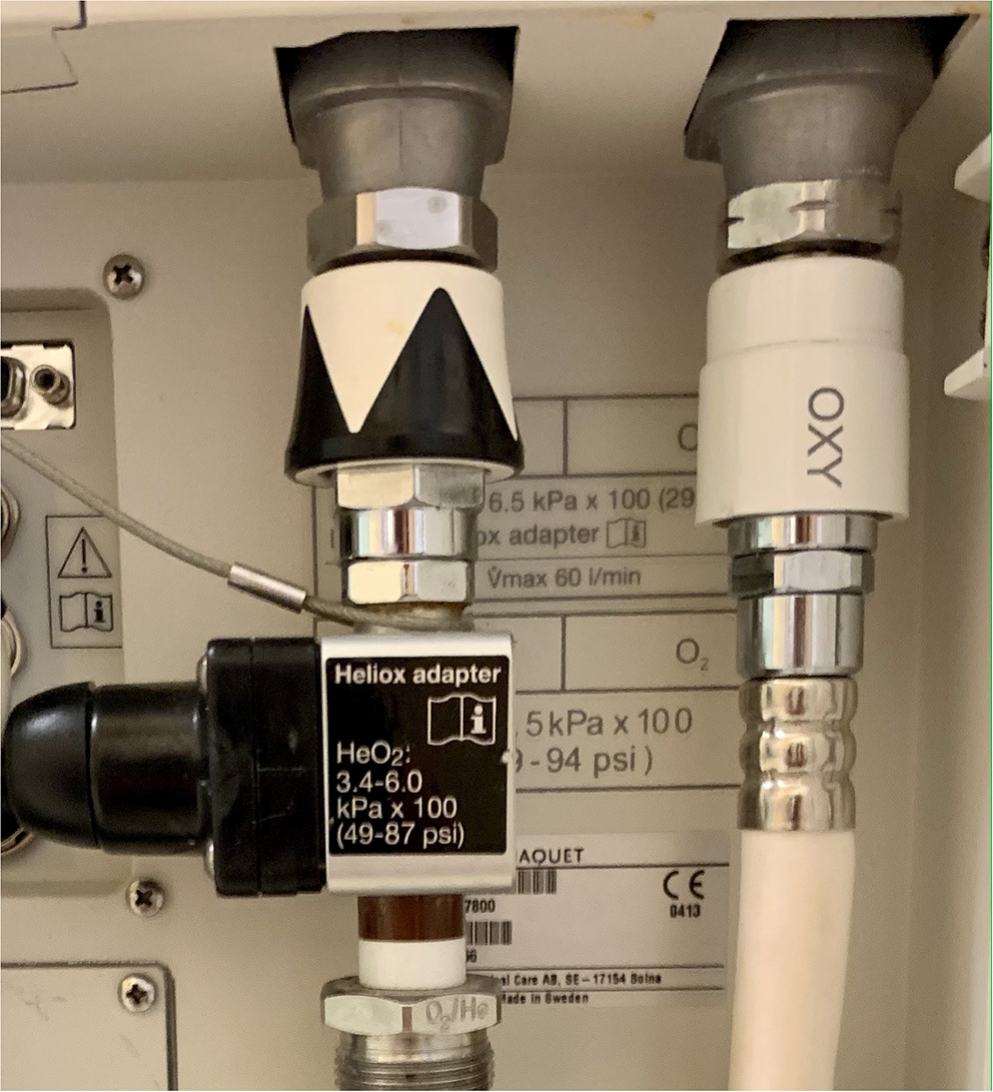
Ventilator air inlet modified for Heliox delivery (Maquet Servo-I, Getinge, Sweden).

For safety reasons pure helium should not be used to avoid the risk of administration of hypoxic mixture. It is possible to either use a premixed helium-oxygen with a set He:O_2_ ratio (e.g., 79:21, 70:30, 60:40) or—preferably—blend 0.21 FiO_2_ Heliox administered *via* modified air inlet of the ventilator (Figure 3) with pure oxygen depending on infants' saturation. However, the latter option requires dedicated equipment. Standard ventilators are calibrated for nitrogen-oxygen mixtures and when Heliox is used instead it may affect the function of the device (e.g., gas mixing, inspiratory and expiratory valve operation, and flow measurement). When helium-oxygen at 80:20 was utilized with ventilators not optimized for this mixture delivered FiO_2_ was lower than set. There were also notable differences between displayed and actually delivered tidal volumes; the scope of this effect was variable depending on the type of the device (52, 53). Another study confirmed unreliable tidal volume measurements at high helium concentration as well as triggering of “high-priority alarm condition that couldn't be disabled” (53).

It is possible to self-adjust conventional ventilators for helium-oxygen mixture delivery according to the guidelines and correction factors available in the literature (7). However, for safety and optimal efficacy it is recommended to use equipment optimized for Heliox delivery by the manufacturer. There are several ventilators available that allow Heliox ventilation in pediatric/neonatal modes, e.g., Event (Event Inspiration Ltd, Ireland), Avea (Vyaire, USA), G5 (Hamilton, USA), and Servo-I (Getinge, Sweden). Precision Flow device (Vapotherm, USA) is also available in a version designed for the high flow therapy. Modern ventilators are equipped with enhanced software and additional hardware elements like a modified connector at air inlet. After Heliox is started the ventilator recognizes the mixture and automatically adjusts set and monitored parameters.

**Figure 4:**
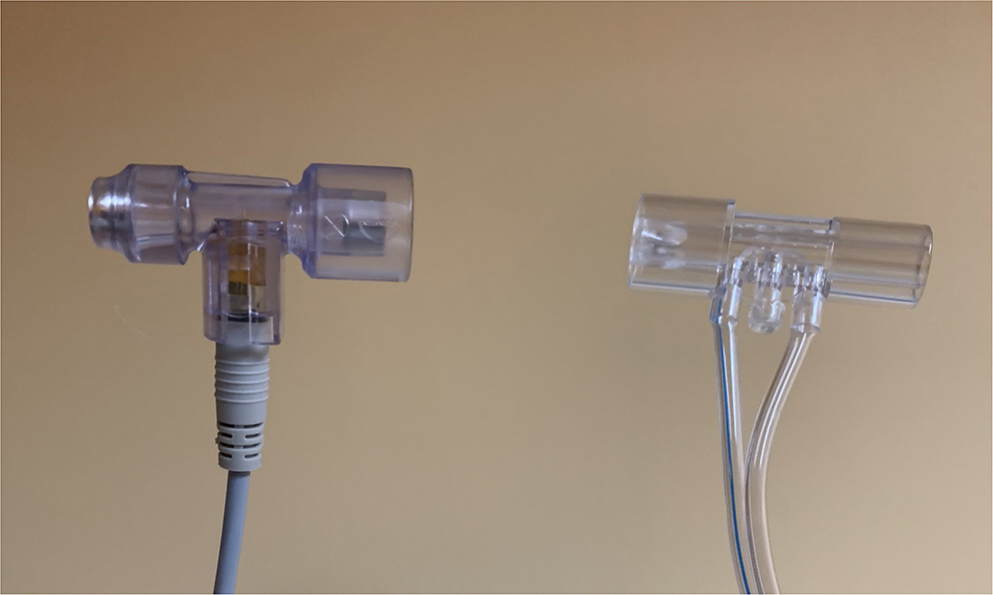
Hot wire sensor and a variable orifice proximal flow sensor.

Analysis of available data suggests that in infants Heliox should be administered with positive pressure in order to observe its beneficial effects. Its application was combined with HFNC, nCPAP, NIPPV, NIV-NAVA and in the intubated neonates (11, 14, 32, 54). Conventional ventilation with Heliox requires a variable orifice proximal flow sensor as standard hot-wire sensors will not provide reliable measurements (Figure 4). Alternatively, heliox can be combined with NAVA on Servo-I both in intubated neonates and non-invasively. Due to the previously mentioned high coefficient of heat conduction appropriate warming and humidification of the gas is particularly important to prevent hypothermia. This can be achieved using standard humidifiers. In theory, Heliox ventilation may be associated with the risk of increased leak but it does not seem to play a significant role in clinical practice.

Effects of Heliox are usually seen within minutes after initiation of the therapy. If this is not the case then the therapy should change to another alternative. Unfortunately, as quick as the respiratory effects of this mixture appear after start of delivery, it will also be noted that after discontinuation they will quickly cease after helium-oxygen is stopped. This phenomenon was observed in several studies in neonates (6, 15, 17). Hence, some authors proposed to consider Heliox as “therapeutic bridge” that allows enhanced support until the primary cause of respiratory failure resolves. Optimal duration of therapy in the newborn remains unknown. After initial improvement with short-term applications of Heliox during MV lasting 1 h prompt deterioration was observed (14, 18, 19). Similar trend was observed after 3 h of NIV-NAVA Heliox, while 12 h of helium-oxygen resulted in reduction of CPAP failure (17). It seems obvious that the length of the Heliox ventilation must be tailored to the individual needs of each patient. Among other issues future studies should aim to identify criteria for the safe weaning. It seems that monitoring changes in oxygen demand and diaphragmatic function (Edi—absolute values and trends) might be helpful in decision making (17). Infants requiring FiO_2_ > 0.5–0.6 are less likely to benefit from Heliox as its effects are lesser with lower helium concentration (48).

## Discussion

Despite convincing theoretical background and encouraging reports there are certain barriers that seem to limit more common use of Heliox. Among them are the above-mentioned technical issues and the need for specialized or modified equipment. Moreover, helium-oxygen mixtures are not available on demand in every country and may require special order which is sometimes a cumbersome and time-consuming procedure. In selected countries (e.g., UK and USA) it is registered as medical gas in other its use may be limited to “experimental” protocol. Usually, it is only provided in cylinders and continuous wall supply is uncommon.

Although in the USA medical gasses represent <1% of total NICU costs (with the exception of inhaled nitric oxide) and Heliox costs are ~20–40% higher than medical oxygen and the price of this mixture may be higher in other parts of the world and pose another important issue (Merritt TA 2021, personal communications). Previous publications reported Heliox to be “costly”—several times more expensive than gasses routinely used for ventilation (2–3 × more than medical air and up to 8–10 × more than oxygen) (7, 48). Heliox price in the UK in 2022 for the “HX” cylinder (1,780 L) is 413 GBP while the same cylinder of medical oxygen (2,300 L) is 17 GBP and medical air G cylinder (3200 L) is 14 GBP pounds (55). A Czech study reported the price of a 50 L cylinder at 200 bar to be 233 EUR vs. 46 EUR for oxygen (also 50 L at 200 bar) (56). As significant differences in Heliox consumption were shown between different ventilators the choice of the device may influence the cost of the therapy (48). Further savings might be achieved with a semi-closed circuit. Jurickova I et al. described a custom-made delivery system that allowed substantial reduction of costs when compared to an open circuit (56).

Another important barrier is associated with the amount and quality of evidence regarding Heliox therapy. There are relatively few studies that involved infants and in most of the cases their sample sizes were small.

In conclusion, Heliox is a safe therapy that offers potential benefits for the newborn infants due to its respiratory effects and perhaps also cardio/neuro-protective properties. Based on available data it seems that this mixture may be helpful in decreasing the risk of NIV failure and reducing the risk of lung injury when MV is necessary. Hence, future investigations are warranted. Although, when planning future trials sufficient sample size and multi-center involvement should be considered.

## Author Contributions

All authors listed have made a substantial, direct, and intellectual contribution to the work and approved it for publication.

## Conflict of Interest

The authors declare that the research was conducted in the absence of any commercial or financial relationships that could be construed as a potential conflict of interest.

## Publisher's Note

All claims expressed in this article are solely those of the authors and do not necessarily represent those of their affiliated organizations, or those of the publisher, the editors and the reviewers. Any product that may be evaluated in this article, or claim that may be made by its manufacturer, is not guaranteed or endorsed by the publisher.
